# The Unresolved Pathophysiology of Lymphedema

**DOI:** 10.3389/fphys.2020.00137

**Published:** 2020-03-17

**Authors:** Syaza Hazwany Azhar, Hwee Ying Lim, Bien-Keem Tan, Veronique Angeli

**Affiliations:** ^1^Department of Microbiology and Immunology, Life Science Institute, Yoon Loo Lin School of Medicine, National University of Singapore, Singapore, Singapore; ^2^Department of Plastic, Reconstructive, and Aesthetic Surgery, Singapore General Hospital, Singapore, Singapore

**Keywords:** lymphedema, pathophysiology, inflammation, adipose tissue, fibrosis

## Abstract

Lymphedema is the clinical manifestation of impaired lymphatic transport. It remains an under-recognized and under-documented clinical condition that still lacks a cure. Despite the substantial advances in the understanding of lymphatic vessel biology and function in the past two decades, there are still unsolved questions regarding the pathophysiology of lymphedema, especially in humans. As a consequence of impaired lymphatic drainage, proteins and lipids accumulate in the interstitial space, causing the regional tissue to undergo extensive and progressive architectural changes, including adipose tissue deposition and fibrosis. These changes are also associated with inflammation. However, the temporal sequence of these events, the relationship between these events, and their interplay during the progression are not clearly understood. Here, we review our current knowledge on the pathophysiology of lymphedema derived from human and animal studies. We also discuss the possible cellular and molecular mechanisms involved in adipose tissue and collagen accumulation during lymphedema. We suggest that more studies should be dedicated to enhancing our understanding of the human pathophysiology of lymphedema to pave the way for new diagnostic and therapeutic avenues for this condition.

## Introduction

The lymphatic system consists of a network of vessels connecting lymphoid organs such as lymph nodes, tonsils, thymus, and spleen. Running parallel to the venous circulation, the primary function of the lymphatic system is to drain excess interstitial fluid leaking out from blood capillaries into the tissue spaces. Other functions include fat absorption in the intestine, immune surveillance, and resolution of inflammation. Lymph fluid transports various antigens and antigen-presenting cells into lymph nodes for immune response. Lymph containing lipids, immune cells, macromolecules, and fluid is first collected by blind-ending initial or capillary lymphatic vessels which in turn empty into larger lymphatic vessels, i.e., the collecting vessels or collectors. Unlike the initial lymphatic vessels, the collecting lymphatics exhibit circumferential smooth muscle cell coverage and luminal valves that propel and maintain unidirectional flow (Tammela and Alitalo, 2010).

Lymphedema is a chronic and progressive disease arising from impaired lymphatic drainage causing the accumulation of interstitial fluid which results in tissue swelling (Rockson, 2001). Lymphatic dysfunction can be caused by genetic abnormalities affecting the lymphatic development and/or function and it typically becomes apparent during infancy, childhood, or adolescence, a condition known as primary lymphedema. Less frequently, primary lymphedema can appear after age 35 and is known as lymphedema tarda (Rockson, 2001; Greene and Maclellan, 2013). The incidence of primary lymphedema is low, affecting 1 in 100,000 people worldwide (Smeltzer et al., 1985). Lymphedema may occur secondary to damage or obstruction of lymphatic vessels due to infectious diseases such as filariasis or trauma, including radiotherapy and surgical removal of lymph node in cancer treatments (Rockson, 2001; Witte, 2001; Greene and Maclellan, 2013). Today, secondary lymphedema is more common, due to increasing cancer rates, affecting 1 in 1,000 persons, whereby 24–49% of cancer patients develop secondary lymphedema after receiving cancer treatment (Karaca-Mandic et al., 2015; Kurt et al., 2016). However, this rate of incidence and prevalence are likely underestimated because lymphedema remains under-recognized and under-documented. Breast cancer-associated lymphedema is the most common form of lymphedema in developed countries followed by sarcoma, gynecologic cancers and malignant melanoma (Rockson, 2018). Tissue swelling in extremities due to interstitial fluid accumulation causes discomfort, restricted range of motion, and decreased quality of life for lymphedema patients. Increased susceptibility to infections, recurrent infections, psychological morbidity, functional disability, skin changes, and malignant transformation are known complications associated with human lymphedema (Greene and Maclellan, 2013).

## Pathophysiology of Lymphedema

Research in the past decades has led to the understanding that the factors causing lymphedema are not solely attributed to lymph and fluid accumulation in the interstitial tissue. It is proposed that its pathophysiology involves a chain of complex and progressive events affecting different tissue compartments. These events are described below although their exact chronological order remains unresolved.

### Adipose Tissue Expansion and Remodeling in Lymphedema

Substantial evidence exists that tissue swelling in lymphedema is due to fat deposition and not just the accumulation of fluid. The presence of excess adipose tissue in the affected limb has been well documented in patients with chronic non-pitting arm lymphedema following breast cancer (Brorson and Svensson, 1997). Our clinical observations show that hypertrophic fat lobules compress and collapse their feeding lymphatic capillaries, resulting in a vicious cycle of fluid and lipid transport disruption, ultimately leading to further fat accumulation in the periphery. A recent study using magnetic resonance imaging (MRI) revealed that fat deposition is not limited to the epifascial compartment (between skin and muscle cells) of the lymphedematous compartment, but is also present in the subfascia (between muscle cells or within muscle cells) which did not change following liposuction (Hoffner et al., 2018b). However, from a clinical perspective, the subcutaneous compartment is the main area of fat accumulation and this fat is amenable to removal with liposuction. Adipose tissue accumulation has also been described in mouse models with lymphatic stasis (Table 1). For example, in mice whose skin lymphatic vessels have been surgically resected, subcutaneous fat deposition and fat thickness increased (Aschen et al., 2012). Similarly, *Chy* mice, a mouse model of lymphedema, due to heterozygous inactivating mutations in the vascular endothelial growth factor receptor 3, developed abnormal subcutaneous fat deposition, especially in edematous subcutaneous adipose tissue close to dysfunctional hypoplastic lymphatic vessels (Karkkainen et al., 2001). In addition, high levels of fat in the tail skin was observed (Rutkowski et al., 2010). In Prox1 heterozygous mice, lymphatic malfunction resulted in lymph leakage and accumulation of adipose tissue, culminating in adult-onset obesity (Harvey et al., 2005). Importantly, *in vivo* restoration of the Prox1 level, specifically in the lymphatic endothelial cell, was sufficient to reverse the lymphatic defects in *Prox1*^±^ mice and ameliorate their obesity phenotype (Escobedo et al., 2016). Together, these mouse and human findings support a relationship between lymphatic dysfunction and adipose tissue accumulation. A detailed histological analysis of the adipose tissue from healthy and patients with lower extremities lymphedema revealed significant differences between the two groups (Tashiro et al., 2017). Macroscopic and ultrasonic examination demonstrated that lymphedema adipose tissues have larger lobules, which are surrounded by thicker collagen matrix and lymph fluid compared to healthy adipose tissue. Healthy adipocytes are non-ruptured and uniformed in size, whereas adipocytes in lymphedema are hypertrophic and highly variable in size (Tashiro et al., 2017).

**TABLE 1:** Animal models of lymphedema.

	Species	Models	Pathophysiology observed	References
Surgical models	Mouse	Tail incision	Increase adipose deposition and adipocyte hypertrophy Macrophages surrounding subcutaneous fat deposits Collagen deposition within subcutaneous fat & collecting lymphatic vessels Changes in immune cell infiltrate Enlarged lymphatic capillaries Loss of function of both initial & collecting lymphatic vessels Increase in VEGF-C locally and systemically	Rutkowski et al., 2010; Zampell et al., 2012a; Cuzzone et al., 2014; Gousopoulos et al., 2016b, 2017; Jun et al., 2017
	Dog Mouse Rat Monkey Pig	Lymph node dissection/removal & radiation	Sustained tissue inflammation Decreased number of regeneration lymphatics Worsened swelling & lymph drainage Reduced fluid transport Dilated lymphatic vessels Increase volume in affected limb Lymph backflow	Knight et al., 1987; Blum et al., 2010; Lahteenvuo et al., 2011; Cuzzone et al., 2014; Wu et al., 2014; Yang et al., 2014; Triacca et al., 2019; Wang et al., 2020
	Mouse Rat	Hind limb incision	Swelling & impaired lymph drainage Insufficient wound healing Reduced lymph transport Dilated lymph vessels Increased fibrosis	Lynch et al., 2015; Jun et al., 2017; Jørgensen et al., 2018
	Rabbit	Ear incision	Dermal backflow Disrupted lymphatic flow Fibrosis Lymphatic vessels dilated and increased in number	Fu et al., 1998; Szuba et al., 2002; Yoon et al., 2003; Fernández Peñuela et al., 2018
Transgenic mouse models	Mouse	*Chy Chy-3*	Swelling in forepaw and hindpaw Higher levels of collagen and fat in skin Devoid of initial lymphatics in dermis Defective lymphatic vessels Decreased lymph flow	Karkkainen et al., 2001; Karlsen et al., 2006, 2012; Dellinger et al., 2007; Rutkowski et al., 2010
	Mouse	*Prox1*^±^	Baseline lymphatic dysfunction Impaired lymphatic transport & leaky lymphatics Impaired immune cell migration Increased inflammation after lymph node removal Defective lymphatic vasculature	Srinivasan et al., 2014; Hespe et al., 2019
	Mouse	K14-VEGFR-3-Ig (sR3)	Lack dermal lymphatics Smaller adipocytes Increased M2 macrophages Lack of podoplanin positive vessels in tail & back skin Regression of developing lymphatics	Mäkinen et al., 2001; Karaman et al., 2015
		FLT4-DTR	Mimic chronic lymphedema Initial swelling resolved temporarily, followed by late onset of lymphedema CD4 + infiltration Inhibition of lmyphagiogenesis Sclerosis of collecting lymphatic vessels	Gardenier et al., 2016

Adipose tissue functions as an energy reservoir either by storing excess nutrients or supplying nutrients to other tissues (Birsoy et al., 2013). It also plays an important endocrine function whereby adipocytes secrete hormones and cytokines, known as adipokines, to regulate energy homeostasis (Rondinone, 2006). In obesity, pathological adipose tissue remodeling occurs when excess nutrients cause adipocytes to expand in size (hypertrophy) or increase in number (hyperplasia) once they have reached their maximum lipid storage capacity. This adipose tissue remodeling leads to dysregulation of adipokine production, metabolic stress, and a low-grade local inflammation through the increased secretion of pro-inflammatory cytokines (TNF-α, IL-6, MCP-1, IL-8) that promote immune cell infiltration and their pro-inflammatory polarization (de Ferranti and Mozaffarian, 2008; Drolet et al., 2008). Adipose tissue hypertrophy in lymphedema is accompanied by adipose remodeling, similar to what occurs in obesity. Increased serum levels of adiponectin and leptin were found in lymphedema patients, likely reflecting expansion of adipose tissue (Zaleska and Olszewski, 2017). Increased adiponectin expression, which correlates with fat accumulation caused by lymph stasis, was also observed in mouse tail surgical model of lymphedema (Aschen et al., 2012) (Table 1). Moreover, IL-6 expression which correlates with adipose tissue depots in obese patients (Mohamed-Ali et al., 1997; Fried et al., 1998) has also been shown to be increased in human and mouse lymphedematous tissues as well as in serum of lymphedema patients (Olszewski et al., 1992; Cuzzone et al., 2014). Increased IL-6 expression in lymphedematous murine tissues is associated with fat deposition, and it is postulated that its role is to regulate adipose tissue homeostasis since blocking its activity limits the expansion of adipose tissue (Cuzzone et al., 2014).

### Fibrosis in Lymphedema

Fibrosis, which is the excessive deposition of extracellular matrix in various organs, potentially leads to their dysfunction. This condition occurs in the extremity lymphedema and is an important pathological change in lymphedema. Histological and immunohistochemical examinations of skin tissues from clinical and experimental lymphedema revealed increased amounts of collagen fibers in the edematous skin (Schirger et al., 1962; Ryan, 1995; Rutkowski et al., 2010; Zampell et al., 2012c; Gardenier et al., 2016; Table 1). Fibrosis in lymphedema is not confined to the dermis, but has also been detected in the subcutaneous tissue including the adipose tissue. Hypertrophic adipocytes exhibit thick fibrous matrix between lobules (Tashiro et al., 2017) in human lymphedema. Collagen accumulation within subcutaneous fat in mouse models of lymphedema was observed (Zampell et al., 2012c; Gardenier et al., 2016). This causes lymphedematous tissue to harden, resulting in non-pitting edema.

Collecting lymphatic vessels have been shown to play a role in lymphedema onset, and studies of lymphedema patients and animal models demonstrated morphological and structural changes in collecting lymphatic vessels including collagen deposition (Mihara et al., 2012; Gardenier et al., 2016). In lymphedema, lymph fluid stasis results in an increase in the pressure within lymphatic vessels. When this process perpetuates, the smooth muscle cells in the lymphatic vessels become slimmer and flattened (Koshima et al., 1996; Ogata et al., 2015) and dermal capillary lymphatic vessels become hypertrophic (Tashiro et al., 2017), causing dermal back flow of lymph fluid. Mihara et al. (2012) elegantly investigated four types of collecting lymphatic vessel changes throughout disease progression in lymphedema patients that began before the onset of lymphedema. Normal type of collecting lymphatic vessels has collagen fibers and smooth muscle cells present in medial layer. Ectasis type is characterized by the dilation of the lymphatic vessel wall, with long and elongated collagen fibers. Contraction type shows the deposition of thick collagen fibers mixed with smooth muscle cells in the medial layer. The thick collagen fibers impair vessel contraction, resulting in loss of function in the collecting lymphatic vessels. Sclerosis type vessels exhibit increased smooth muscle cells and collagen fibers and a loss in their ability to transport lymph fluid, causing excessive lymph leakage (Mihara et al., 2012). These changes in collecting vessels are consistent with previous findings showing decreased lymphatic vessels contractility in human lymphedema (Olszewski, 2002; Modi et al., 2007). In addition, fibrosis in the skin and subcutaneous tissue may worsen lymphatic vessel dysfunction by directly inhibiting lymphatic endothelial cell proliferation and preventing the sprouting and branching of new lymphatic vessels. This is supported by findings in mice and rat models demonstrating that fibrosis negatively regulates lymphatic flow and lymphangiogenesis, which in turn aggravate swelling, fluid transport and lymph drainage (Lynch et al., 2015). However, when fibrosis was inhibited, lymphatic vessel repair and transport were improved, slowing down the progression of lymphedema (Avraham et al., 2009, 2010; Savetsky et al., 2014).

In contrast to our knowledge on the deleterious effect of increased collagen deposition on lymphedema, little information is available on the collagen fiber structure and spatial organization in lymphedema. Two recent studies on human and mouse lymphedema tissues using multi-photon microscopy revealed changes in the spatial organization of collagen network, leading to irreversible structural damages (Wu et al., 2011; Kistenev et al., 2019). However, the possible consequences of these changes on lymphatic function and lymphedema remains to be elucidated.

### Skin Changes

In the later stages of lymphedema progression, skin changes such as hyperkeratosis may occur together with fibrosis of dermis, subcutaneous tissue, and muscular fascia (Daroczy, 1995; Domaszewska-Szostek et al., 2016). As the disease progresses, skin indurated developing a leathery texture and is more prone to recurrent infections, wart formation, cellulitis, warts, ulceration, fissures, and in rare cases, cutaneous angiosarcoma (Sinclair et al., 1998; Grada and Phillips, 2017).

## Potential Mechanisms for Tissue Transformation in Lymphedema

The mechanism of adipose tissue and fibrosis in lymphedema remains unclear, and several studies especially in mouse model of lymphedema have revealed potential factors in these processes.

### Role of Lymph Stasis and Deposited Lipids

In lymphedema, the affected tissue becomes suffused with lymph as a result of lymphatic obstruction or insufficiency. Studies by G. Oliver’s group in *Prox1*^±^ mice provided the first evidence that lymphatic leakage can promote adipose tissue hypertrophy and adipogenesis (Harvey et al., 2005). They showed that fat accumulates near leaky mesenteric lymphatic vessels in *Prox1*^±^ mice, and the egressed fluid induces *in vitro* the differentiation of adipocyte (Harvey et al., 2005). One indication of adipogenesis is the increased expression of fat differentiation markers demonstrated in a tail model of lymphedema (Aschen et al., 2012). Stagnant lymph contains various factors including proteins and lipids. In a follow-up study, G. Oliver’s group demonstrated that the lipid fraction within the leaking fluid is the adipogenic factor, although no significant differences in lipid composition of lymph from WT and *Prox1*^±^ mice were noted. Lymph from both groups promoted the differentiation of preadipocytes into mature adipocytes (Escobedo et al., 2016). This finding is consistent with a previous work showing that mesenteric lymph, or more specifically, chylomicron isolated from the lymph, supports the differentiation of adipocyte precursors (Cambon et al., 1998). Furthermore, free fatty acids, which are abundant in lymph, promoted adipogenesis *in vitro* (Escobedo et al., 2016). Cholesterol is another lipid component that may deposit in tissue from lymph stasis. Lipoproteins including low-density (LDL) and high-density (HDL) lipoproteins play a critical role in lipid transport in lymph, as well as in blood (Randolph and Miller, 2014). The transport of cholesterol conjugated to HDL from peripheral tissues back to the systemic circulation is known as reverse cholesterol transport (RCT) and critically depends on efficient lymphatic transport. In experimental models, the surgical disruption of lymphatic channels impairs the return of cholesterol to the systemic circulation from a transplanted artery (Martel et al., 2013) and the skin (Lim et al., 2013). Notably, adipose tissue is the major site of cholesterol storage and alterations in cholesterol balance in adipocytes can modulate metabolic and pro-inflammatory adipose tissue functions (Chung and Parks, 2016). This raises the possibility that RCT may be compromised in lymphedema as a consequence of impaired lymphatic drainage. The resultant cholesterol accumulation in the affected limb may in turn contribute to adipose remodeling. This phenomenon needs to be demonstrated experimentally. From a clinical perspective, the above lipid factors in lymphedematous tissue, and correspondingly, their levels in the blood have not yet been clearly studies.

Traditionally, it is believed that high protein content in the interstitial tissue induces fat deposition and fibrosis. A study in the K14-VEGFR3-Ig transgenic lymphedema mouse model revealed that lymph stasis *per se* may not be sufficient to induce these tissue changes (Markhus et al., 2013). Clearly, other factors and events evolving around lymph stasis trigger these pathological changes in the lymphedematous tissues, explaining why the majority of the patients develop lymphedema months to years after the initial injury (Petrek et al., 2001).

### Role of Inflammation

It is clear from both experimental and clinical studies that inflammation is a critical player in the pathophysiology of lymphedema (Ly et al., 2017). The first demonstration that lymphedema in rats leads to chronic inflammatory response (Gaffney and Casley-Smith, 1981), together with the later identification of inflammatory genes associated with several symptoms in human lymphedema (Fu et al., 2016), has fueled more research this area. One of the well-characterized manifestations of the inflammatory reaction associated with lymphedema is the infiltration of inflammatory cells in the edematous tissues. Studies by the group of B. J. Mehrara and M. Detmar showed in clinical and experimental lymphedema that the majority of the cells that accumulate chronically in lymphedematous tissues are CD4^+^ T cells and that they contribute to the pathological changes including fibrosis (Avraham et al., 2010, 2013; Zampell et al., 2012c; Savetsky et al., 2014; Gousopoulos et al., 2016b). Further phenotypic characterization of the infiltrating CD4^+^ T cells revealed that there is a mix of Th1, Th2, and regulatory T cells (Avraham et al., 2013; Gousopoulos et al., 2016a). Notably, the blockade of Th2 differentiation but not Th1 differentiation was effective in preventing the development of lymphedema, and in established cases, treating it (Avraham et al., 2013; Ly et al., 2019). Similar results were obtained when the pro-fibrotic cytokines and growth factors i.e., IL-4, IL-13, and TGF-β, produced by Th2 cells were blocked (Avraham et al., 2013; Savetsky et al., 2015). In contrast to the pathogenic role of Th2 cells in lymphedema, T regulatory (Treg) cells seem to limit the pathological changes in lymphedema. Indeed, the depletion of Treg cells exacerbates edema and fibrosis and is associated with increased infiltration of immune cells with a mixed Th1/Th2 cytokine profile (Gousopoulos et al., 2016a). Conversely, expansion of T regulatory cells significantly reduced lymphedema development by attenuating the tissue inflammation in lymphedema (Gousopoulos et al., 2016a). However, these cells may also participate to the local immune suppression observed in lymphedema, which is consistent with the recurrence of soft tissue infections observed in this disease (Sharkey et al., 2017; Garcia Nores et al., 2018). Collectively, these findings suggest targeting T cells as a potential novel therapeutic strategy for lymphedema.

Macrophages can serve multiple functions including regulation of lymphatic vessels (Kataru et al., 2009), inflammation, immunity, and tissue repair (Ginhoux and Jung, 2014), which are all functions relevant to lymphedema progression. Accumulation of macrophages has been detected in lymphedema. However, macrophages have been shown to serve opposing functions. Macrophages have been commonly classified into alternatively activated macrophages (M2) or classical (M1) phenotype, with repair and pro-inflammatory functions, respectively. However, this classification is simplistic and may not represent the entire spectrum of macrophage phenotypes and their corresponding functions *in vivo* (Ginhoux and Jung, 2014). Depletion of macrophages in the mouse tail surgery model significantly promotes fibrosis (Zampell et al., 2012b; Ghanta et al., 2015). Macrophages that exhibit a M2 phenotype may mediate this anti-fibrotic function through the regulation of CD4^+^ T cell accumulation and Th2 differentiation (Ghanta et al., 2015; Savetsky et al., 2015; Shin et al., 2015). In healthy adipose tissue, M2 macrophages are dominant. In adipose tissue of obese individuals, the number of M2 macrophages decreased while M1 macrophages appear to be more frequent. In line with these observations in obesity, flow cytometry analysis of adipose tissue-derived cells from healthy and lymphedema subjects showed that there is an imbalance between M1 and M2 macrophages, where M2 macrophages decreased in number in lymphedema adipose tissues compared to healthy controls (Tashiro et al., 2017). Notably, M1 macrophages in obese adipose tissue have been shown to localize predominantly to dead adipocytes to form crown-like structures and to scavenge residual lipid and debris from necrotic adipocytes (Cinti et al., 2005). The report by Tashiro et al. reveals that the accumulation of M1 macrophages in adipose tissue of lymphedema patient was rarely associated with crown-like structures (Tashiro et al., 2017). In the mouse tail model, lymph stasis is associated with the infiltration of F4/80^+^ macrophages, which accumulate around the expanded subcutaneous fat (Zampell et al., 2012c). Whether this infiltration is a prelude to crown-like structure formation can only be confirmed by the presence of adipocyte necrosis and the M1 macrophage phenotype. Therefore, further investigations are warranted to study the formation of crown-like structures in lymphedema-associated adipocyte remodeling and its significance. Macrophages may also control adipose tissue remodeling through the production of IL-6 that is a key factor in chronic inflammation and adipose metabolism (Scheller et al., 2011). In addition, macrophages may play a role in lymphedema by controlling lymphangiogenesis through the production of vascular endothelia growth factor-C, since depletion of macrophage, in established lymphedema, decreases lymphatic transport activity and VEGF-C expression (Zampell et al., 2012b; Ghanta et al., 2015). Finally, macrophages may improve lymph stasis through the upregulation of lymphatic pumping activity by modulating the expression of inducible nitric oxide synthase (Liao et al., 2011; Scallan et al., 2016). Altogether, these findings suggest a complex role for macrophages in the pathophysiology of lymphedema. This diversity of functions may depend on the stage of the disease, its anatomical location (upper versus lower extremity), and the type of macrophage population.

Clinical and animal studies show that inflammatory genes are upregulated in lymphedema (Foldi et al., 2000; Lin et al., 2012; Leung et al., 2014). Importantly, the expression of pro-inflammatory genes, such as TNF-α and IFN-γ, were decreased after complete decongestive treatment. Transcriptional profiling of lymphedematous tissues in a mouse tail model revealed the upregulation of genes involved in acute inflammation, immune response, fibrosis, and wound healing (Tabibiazar et al., 2006). The authors hypothesize that leukotrienes produced by 5-lipoxygenase (5-LO) have a potential role in the pathogenesis of the disease. Human lymphedema patients exhibit increased levels of plasma leukotriene B4 (LTB4)(Tian et al., 2017). In mice treated with LTB4 antagonist, edema is reversed, together with improvement in lymphatic function and skin pathological changes (Tian et al., 2017). Ketoprofen is a NSAID drug with a dual anti-inflammatory mechanism of action, including inhibition of the 5-LO pathway (Rajic et al., 2010). Interestingly, the systemic treatment with ketoprofen of mice with established lymphedema reverses the disease and histopathological changes (Nakamura et al., 2009). The inhibition of the 5-LO pathway account for the therapeutic effect of ketoprofen. Together these preclinical results led to the clinical pilot study to evaluate the potential therapeutic effect of ketoprofen to ameliorate human lymphedema. This exploratory study demonstrated the beneficial effect of targeted anti-inflammatory therapy with ketoprofen in lymphedema patients, as shown by the reduction of skin thickness and amelioration of histological changes (Rockson et al., 2018). It remains to be seen whether this treatment reverses fibrosis, adipose tissue deposition and is long-lasting.

## Diagnosis and Assessment of Lymphedema and Associated Tissue Changes

Lymphedema is diagnosed clinically and classified in four stages according to International Society of Lymphology (ISL). Stage 0 is latent, and despite impairment of lymph transport, swelling is not evident. Stage I is characterized by early accumulation of fluid, and elevating the affected limb may subside swelling. Pitting (indentation remains when a finger is pressed onto affected area) may also occur in Stage I. Pitting is more evident in Stage II, as swelling increases and will not subside from limb elevation alone. As it progresses to late Stage II, pitting may or may not occur due to onset of fibrosis. Stage III lymphedema, also known as lymphostatic elephantiasis, is advanced lymphedema with pitting absent.

Methods to diagnose lymphedema have primarily focused on the detection of edema, lymphatic vessel transport, and lymph flow, until the recent advances in our knowledge of disease pathophysiology prompted the development of methods to assess tissue transformation including fibrosis and fat deposition (O’Donnell et al., 2017). These novel approaches may be more effective modalities to monitor the progression of the disease and the response to treatments that are, to our knowledge, still lacking in the clinic. Analysis of lymphatic vessel structure and transport is carried out by direct or indirect lymphography. Direct lymphography is the injection of contrasting agents into lymphatic vessels (Kinmonth, 1952). Because of the risk of damaging lymphatic vessels, this method has been replaced overtime by indirect lymphography based on the introduction of radiolabeled contrast agents injected into soft tissue that will penetrate the lymphatic vessels allowing their analysis (Cambria et al., 1993). However, there is a lack of standardization, due to the different isotopes used. More recently, infrared fluorescence imaging of lymphatic vessels using indocyanine green dye has enabled the visualization of fine lymphatic vessels and has been used to diagnose and grade lymphedema (Unno et al., 2010). The latest imaging modality is photoacoustic lymphangiography, which provides high resolution imaging of lymphatic vessels and veins (Kajita et al., 2020).

Methods aiming at measuring and recording increase in limb volume include water displacement, optoelectronic perometry, bioelectrical impedance and circumferential measurements (Rincon et al., 2016). However, these methods are not ineffective in detecting early lymphedema.

Non-invasive methods such as ultrasonography, MRI, computerized tomography, and dual-energy X-ray absorptiometry are able to detect skin tissue changes such as tissue density variations, fluid accumulation, fibrosis, and fat components. Although these methods have been used and are able to provide information on the lymphoedematous tissue, they are expensive and complex and present some potential hazards (Brorson et al., 1998; Ward, 2011). Lately, SkinFibroMeter has shown promising results for the assessment of skin stiffness in human lymphedema using a special three-dimensional computational finite element to analyze the biomechanical response of skin tissue to external force (Sun et al., 2017). As discussed above, multi-photon microscopy allows the analysis of collagen structure and may also be used for lymphedema diagnosis as this modality has been used for *in vivo* assessment of human skin aging and photoaging (Lin et al., 2005; Koehler et al., 2006).

## Management of Lymphedema by Multi-Modalities Treatment

There is yet no cure for lymphedema. The current treatments are multi-modality and aim to reduce the swelling and discomfort of the affected extremity in lymphedema patients. One treatment option to manage early stage lymphedema is decongestive therapy, which includes manual lymphatic drainage (MLD), compression bandaging, exercise, skin care, and compression garments. Surgical therapies are indicated for stage I onward, with the modalities being broadly classified into lymphatic reconstructive procedures and excisional procedures (Raju and Chang, 2015). Excisional procedures can produce drastic reductions in limb girth, but may be complicated by unstable scars and poor aesthetic appearance. Lymphatic reconstructive procedures such as lymphovenous bypass and lymphatico-lymphatic anastomosis are useful acute surgical decongestive therapies and may be accomplished stage-wise under local anesthesia. The creation of a peripheral connection between lymphatic and venous systems to treat lymphedema was described as early as the 1960s (Olszewski et al., 1968), but it was not until the 2000s (Koshima et al., 2000), with the introduction of refined instruments and techniques, that lymphovenous bypass gained traction worldwide. The advantages of the technique are that it is minimally invasive and is effective in draining lymphatic fluid immediately. Lymphovenous bypass is coupled with compressive therapy. However, fibrosis at late stages of lymphedema may limit the efficiency of this surgical intervention by compromising the function of the remaining lymphatic vessels that become no longer suitable (Suami and Chang, 2010). Timing of intervention is therefore important, and it is proposed that surgical interventions have better outcomes when performed at earlier stages of the disease (Becker et al., 2006).

In recent years, vascularized lymph node transfer has become a rapidly emerging method of lymphatic reconstruction shown to lead to lymphatic regeneration (Tan et al., 2016). The implanted lymph nodes create new channels and pathways through which fluid drains. Lymphangiogenesis is mediated by vascular endothelial growth factor C (Viitanen et al., 2013). When new channels sprout from the transferred lymph node connected to a peripheral artery and vein functions, it becomes a vascularized lymphaticovenous bypass “relay station.” With better understanding of the vascular anatomy of lymph nodes, surgeons are able to safely harvest lymph nodes from the groin, head, and neck region and abdomen for transfer as lymph node flaps (Gould et al., 2018). From clinical observations, it takes about 2 years for new lymphatic channels to form and be functional. The disadvantage of the technique is donor site morbidity resulting in lymphedema.

The observations that tissue swelling in lymphedema is due to fat deposition have led to the development of liposuction for the treatment of this disease (Brorson et al., 2006, 2009; Damstra et al., 2009). Our clinical observations show that hypertrophic fat lobules compress and collapse their feeding lymphatic capillaries, resulting in a vicious cycle of fluid and lipid transport disruption, ultimately leading to further fat accumulation in the periphery. Conservative forms of surgery such as lymphaticovenous bypass promote clearance of lymphatic fluid and the lipids therein, but are ineffective for large volume fat clearance. Today, with better understanding of lymphatic anatomy and the path of lymphatic channels, surgeons perform selective liposuction where fat is removed with minimal disruption of lymphatic channels (Brorson, 2016). The indication for liposuction is fat hypertrophy in the affected extremities as shown on MRI. Proponents of liposuction demonstrate good volume reduction and no recurrence after 5 years (Hoffner et al., 2018a). The risks of liposuction include blood loss, hematoma, contour irregularity, and skin necrosis (Chen et al., 2019). These patients need to be on lifelong compression garments.

## Conclusion and Future Directions

**FIGURE 1:**
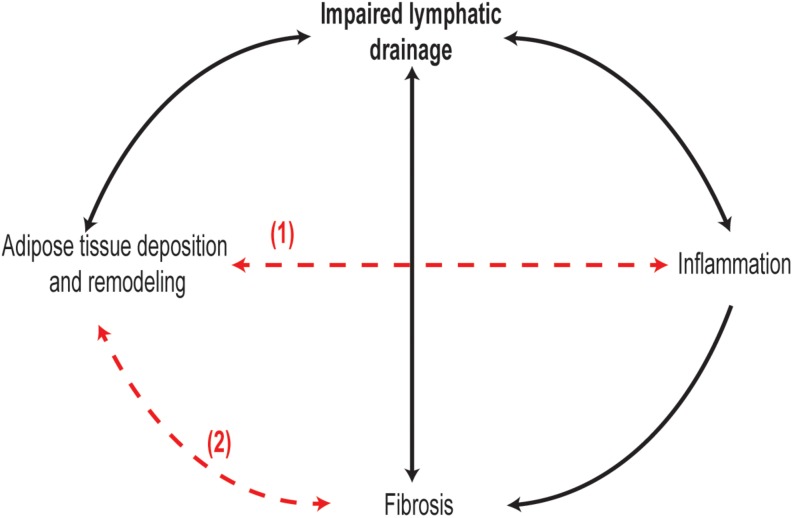
Interplay and chronology between lymphedema pathological changes are not well understood. It is evident in clinical and animal models of lymphedema that inflammation, adipose tissue remodeling, and fibrosis in skin and lymphatic vessels occur as lymphedema progresses. In particular, chronology of adipose tissue remodeling between lymphedema stages remains unexplored. Moreover, it is not known if adipose tissue remodeling (1) and inflammation are two separate or synchronized events, and whether adipose tissue remodeling in lymphedema can promote fibrosis (2) as observed in obesity.

In conclusion, lymphedema is characterized by several pathophysiological events, including lymph stasis, lymphatic vessel remodeling and dysfunction, inflammation, adipose tissue deposition, and fibrosis. However, the exact sequence of these events and their interplay during the development and progression of lymphedema are far from being well described (Figure 1). Much of the recent knowledge in the pathophysiology of lymphedema is derived from animal models of lymphedema, especially mouse models. Most lymphedema animal models are acute, whereby swelling occurs immediately after lymphatic injury and resolves within weeks, with few exceptions such as the FLT4-DTR mouse which develops a more prolonged state of lymphedema with human pathological features (Gardenier et al., 2016; Table 1). In contrast, lymphedema in humans is chronic and lifelong, developing a few months or years after surgery. Therefore, animal models poorly stimulate the onset human lymphedema development and progression. Due to this limitation, not all observations in animal models of acute lymphedema may be extrapolated to humans. More human studies are needed with particular focus on the types of tissue changes across the stages of lymphedema. A better understanding of the pathophysiology of lymphedema and its cellular and molecular mediators will pave the way for novel therapeutic approaches for this chronic and debilitating condition.

## Author Contributions

VA, HL, B-KT, and SA contributed to the manuscript. SA and HL generated the figure and table.

## Conflict of Interest

The authors declare that the research was conducted in the absence of any commercial or financial relationships that could be construed as a potential conflict of interest.
